# Safety and Long-Term Efficacy of Intravitreal rtPA, Bevacizumab and SF_6_ Injection in Patients with Submacular Hemorrhage Secondary to Age-Related Macular Degeneration

**DOI:** 10.3390/jcm14103449

**Published:** 2025-05-15

**Authors:** Peter Wolfrum, Elsa Wilma Böhm, Simon König, Katrin Lorenz, Bernhard Stoffelns, Christina A. Korb

**Affiliations:** Department of Ophthalmology, University Medical Center of the Johannes Gutenberg-University Mainz, Langenbeckstr. 1, 55131 Mainz, Germany

**Keywords:** retina, CNV, anti-VEGF, AMD, submacular hemorrhage

## Abstract

**Purpose:** Acute submacular hemorrhage (SMH) is a vision-threatening complication common in patients affected by age-related macular degeneration (AMD). This study evaluates safety, long-term clinical outcomes and associated treatment factors following intravitreal triple injection of recombinant tissue plasminogen activator (rtPA), SF_6_ gas, and Bevacizumab due to acute SMH secondary to AMD. **Methods:** A retrospective analysis on patients who received treatment between January 2014 and December 2020 (*n* = 37) was conducted. Visual acuity (VA), central retinal thickness (CRT), central retinal volume (CRV), and axial pigment epithelial detachment height were analyzed at baseline (B), 4 weeks after triple injection (FU1), after the following anti-VEGF injection series (FU2), after 1 year (FU3), after 2 years (FU4), and at the final follow-up examination after 4.4 ± 1.6 years (FU5). Further, treatment courses and clinical outcomes were compared to a patient cohort treated for exudative AMD without prior SMH. Furthermore, an explorative data analysis on final VA was conducted, and adverse events following triple therapy were investigated. **Results:** Triple injection was performed on average 5.6 ± 5.7 days after onset of symptoms. Patients received 16 ± 3 additional intravitreal anti-VEGF injections due to persistent macular edema over the subsequent 2 years. Significant improvements were observed at FU1 in VA (*p* < 0.001), CRT (*p* = 0.005), and CRV (*p* = 0.007), as well as at FU2 in axial PED height (*p* < 0.001), with all improvements being stable until final follow-up examination. In the group comparison, patients with SMH demonstrated significantly worse functional and anatomical outcomes at 24 months except for the 24-month CRT, and patients on average received more intravitreal injections. Five of 37 patients (13.5%) experienced a retinal pigment epithelial tear following triple injection. Final VA correlated positively and significantly with FU1 VA, while no correlation was observed with baseline VA, the size or height of SMH, or the number of additional anti-VEGF injections. **Conclusions:** Triple injection constitutes a simple and effective therapy with long-term functional and anatomical improvements following treatment due to SMH, although patients have an increased risk for RPE tears. The 4-week postoperative VA following triple injection was predictive for long-term visual function.

## 1. Introduction

Acute submacular hemorrhages (SMH) can arise from a variety of underlying conditions, including both traumatic and non-traumatic causes. These include direct ocular trauma; arterial macroaneurysms, which can lead to spontaneous hemorrhages due to vascular instability; high myopia, which contributes to degenerative retinal changes and an increased susceptibility to bleeding; or in some cases, an idiopathic condition without an identifiable cause. Additionally, the most common causative factor for spontaneous SMH is age-related macular degeneration (AMD) [1,2]. An increased risk of developing SMH secondary to AMD has been associated with the intake of antiplatelet and anticoagulants as well as uncontrolled arterial hypertension [3,4].

SMH can result in severe and irreversible damages to photoreceptors within hours to days. The damage is thereby primarily driven by iron toxicity, a disruption of essential nutrient and oxygen delivery, as well as mechanical damages due to blot clots, and ultimately, the formation of fibrotic scars [1,5,6]. The natural history of patients suffering from SMH secondary to AMD is often characterized by a poor visual prognosis, particularly when the hemorrhage involves the foveal region. As described in a retrospective study of patients affected by acute SMH secondary to AMD, Scupola et al. observed that over a two-year follow-up period, the already poor mean baseline visual acuity (VA) of 20/240 showed further deterioration in 80% of affected eyes, with a markedly reduced final VA of 20/1250 [7].

In the last 20 years, various treatment approaches for the management of acute SMH have been proposed, including the administration of recombinant tissue plasminogen activator (rtPA) intravitreally or into the subretinal space. rtPA constitutes a thrombolytic enzyme, which facilitates the breakdown of blood clots by converting plasminogen into plasmin [8].

The intravitreal injection of rtPA is typically performed in combination with the administration of an expansile gas, such as sulfur hexafluoride (SF_6_), followed by a prone position to induce a pneumatic displacement of the hemorrhage out of the foveal region. Therapeutic outcomes have thereby been further improved by the co-application of anti-VEGF agents [9,10,11]. The subretinal injection of rtPA, on the other hand, in combination with a pars plana vitrectomy (PPV) and retinotomy, can also be combined with intravitreal injections of anti-VEGF agents or expansile gasses [12,13].

Even though the intravitreal injection of rtPA is generally considered as a less invasive procedure compared to the subretinal injection, both treatment modalities are associated with a variety of potential adverse events. Hereby, the most frequent complication includes a significant rise in intraocular pressure (IOP), while in other cases, severe ocular conditions include the development of retinal pigment epithelium (RPE) tears, retinal detachments, or the onset of endophthalmitis.

To this day, no distinct treatment guideline exits in Germany, primarily due to the missing of randomized controlled trials as well as the limited number of studies investigating the outcomes of patients affected by SMH. While the existing literature primarily focuses on the short-term clinical outcomes of patients treated for SMH, there is considerably less data available regarding the efficacy of long-term treatments. In this study, we therefore investigated safety as well as long-term functional and morphological outcomes of patients affected by acute SMH secondary to AMD who received an intravitreal rtPA, SF_6_, and Bevacizumab injection. Additionally, as part of a group comparison, treatment courses and clinical outcomes were compared to patients treated for exudative AMD without prior SMH. 

## 2. Methods

### 2.1. Ethical Approval and Patient Consent

In this study, we conducted a retrospective investigation by reviewing patient records from daily clinical routine. The data used for analysis was further anonymized. Since no patient was prospectively involved and no third party was granted access to original data, ethical approval for this study was not required and no informed consent was obtained, thereby adhering to the regulations of section 36–37 of the Rhineland Palatinate State Hospitals Act (“Landeskrankenhausgesetz”), as well as the Declaration of Helsinki and its later adjustments.

### 2.2. Selection of Patients and Exclusion Criteria

All patients who received triple injection due to SMH secondary to AMD at the Department of Ophthalmology, Mainz University Medical Center, between January 2014 and December 2020 were reviewed and considered for inclusion, and their final treatment outcomes were further monitored until November 2024. Both treatment-naïve patients as well as patients having received prior anti-VEGF therapy were thereby included. In clinical routine, slit lamp examination was used to diagnose acute SMH, which was again confirmed by macular centered optical coherence tomography (OCT).

The exclusion criteria were the absence of relevant data and incomplete patient records as well as the presence of retinal comorbidities leading to SMH or choroidal neovascularization other than age-related macular degeneration, such as diabetic retinopathy (DR), a history of vascular occlusion, pathological myopia, or central serous chorioretinopathy.

For further group comparison, a randomly selected treatment-naïve patient cohort, which was treated for exudative AMD between 2014 and 2024 without prior SMH, was chosen. The selected comparison group showed no statistically significant difference in age and did not meet any of the previously mentioned exclusion criteria.

### 2.3. Treatment Procedure and Subsequent Anti-VEGF Therapy

All patients were treated as inpatients and received an intravitreal triple injection consisting of 75 μg rtPA (Actilyse^®^, Boehringer Ingelheim, Germany), 1.25 mg Bevacizumab (Avastin^®^ 25 mg/mL), as well as 0.3 mL (100%) sulfur hexafluoride (SF_6_) gas via the pars plana as well as a subsequent anterior chamber paracentesis. In all cases, patients had been adequately informed about the off-label intravitreal use of rtPA prior to triple injection. Following the procedure, patients were instructed to maintain a prone position for the next 48 h. On the first and second postoperative day, a slit lamp examination was performed, and the IOP was checked.

Four weeks after the procedure, patients were again reevaluated, and a subsequent anti-VEGF injection series of 3 injections, administered every 4 weeks, was applied. If intra or subretinal fluid persisted, patients were treated with additional anti-VEGF injection series according to a pro re nata therapy scheme with triple injections, in compliance with the treatment guideline of the German Ophthalmological Society [14]. During the subsequent course of treatment, patients received Aflibercept, Ranibizumab, Faricimab, or Bevacizumab.

### 2.4. Outcome Measures and Follow-Up Period

Epidemiological data was collected from each patient, including age, sex, laterality, lens status, prior treatment history, and time since occurrence of symptoms. Further, we analyzed functional and anatomical changes, following the initial triple injection. As functional measure, the decimal VA was collected and transferred into the ETDRS letter score for better comparison of longitudinal changes (conversion of VA according to Beck et al. [15]). For the morphological assessment, high-resolution spectral-domain optical coherence tomography (SD-OCT) volume scans (20° × 20°) centered on the macular region were previously performed during clinical routine (Spectralis™, Heidelberg Engineering, Heidelberg, Germany). Anatomical measures included the automatically measured average 1-mm^2^ central retinal thickness (CRT) and 1-mm^2^ central retinal volume (CRV) as well as the height of the highest axial pigment epithelial detachment within the central 1-mm^2^ area, which was measured manually. The size of the submacular hemorrhages was measured using fundus photography and expressed relative to the size of the optic disc. Further, for the conducted group comparison, the ETDRS letter score, CRT, axial PED height, and number of injections were similarly analyzed in the patient cohort without prior SMH. To investigate potential associated treatment factors of the long-term visual function, an explorative data analysis was performed. The outcome measures were collected at baseline (B), 4 weeks after triple injection (FU1), 4 weeks after the first anti-VEGF injection series (FU2), after 1 year (FU3), after 2 years (FU4), and at the final follow-up visit (FU5), which was conducted on average 4.4 ± 1.6 years after triple injection, with a follow-up range from 2.8 to 6.9 years.

Finally, patient records were reviewed for adverse events following triple injection, such as an elevation of IOP, vitreous hemorrhages, RPE tears, endophthalmitis, or retinal detachments.

### 2.5. Statistical Analysis

For the statistical analyses, SPSS (Version 27; Armonk, NY, USA) was used. A generalized linear mixed model including a Bonferroni correction was applied to compare the mean values of the ETDRS letter score, CRT, CRV, and axial PED height between baseline and follow-up examinations. Regarding the group comparison, clinical outcomes were compared between treatment-naïve patients with SMH and the treatment-naïve comparison group without SMH by performing Wilcoxon–Mann–Whitney tests or independent t-tests, depending on the normality of data distribution. To evaluate the influence of age, time (measured in days) since onset of symptoms, the size of the subretinal hemorrhage (measured in disc areas), baseline VA, FU1 VA, and baseline CRT on final visual acuity, a Spearman correlation was performed. Statistical significance was considered as *p* < 0.05.

## 3. Results

We included 37 eyes of 35 patients (16 females) who received triple injection due to SMH. Five eyes had previously received treatment with anti-VEGF injections due to neovascular age-related macular degeneration (nAMD), with an average of 21.8 ± 14.3 prior intravitreal injections, while the remaining 32 eyes were treatment naïve. The mean age of the study population was 78 ± 6.8 years, with treatment-naïve and previously treated patients exhibiting comparable age profiles (77.9 ± 6.9 years and 79.7 ± 6.6 years, respectively). Treatment was conducted on average 5.6 ± 5.7 days after the first onset of symptoms and the size of the SMH measured on average 11.4 ± 9.4 disc areas. The complete baseline characteristics of the patient cohort is summarized in Table 1.

**Figure 1:**
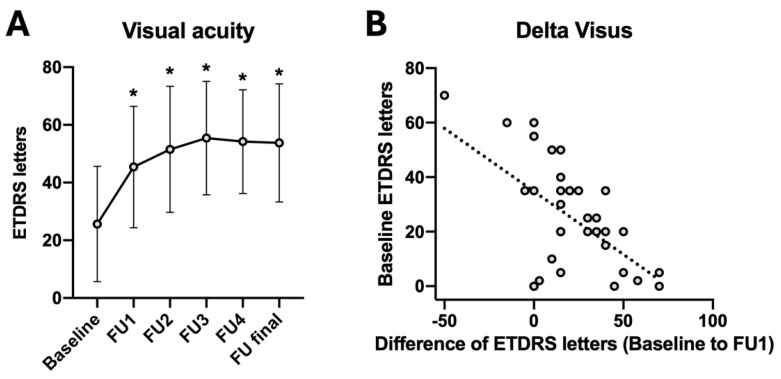
Visual function changes. Significant improvement of the mean visual acuity (ETDRS letters), 4 weeks after triple therapy (**A**). Significant association of ETDRS letter score change (Baseline to FU1) and baseline ETDRS letter score (correlation coefficient: −0.559; *p* < 0.001) (**B**). Note: FU1: 4 weeks follow-up after intravitreal triple injection; FU2: after first injection series; FU3: 1 year; FU4: 2 years; FU final: Last follow-up examination (after 4.4 ± 1.6 years). * Statistically significant in comparison to baseline measurement; *p* < 0.05.

An immediate and statistically significant improvement of visual acuity was observed, with the ETDRS letter score increasing from 25.6 ± 20.0 letters at baseline to 45.4 ± 21.0 letters at FU1 following triple injection (*p* < 0.001). The improvements remained stable until the final follow-up, with an average gain of + 28.2 letters noted. Further, a statistically significant negative correlation was observed between the baseline ETDRS letter score and the change in ETDRS letters from baseline to FU1 (correlation coefficient: −0.559; *p* < 0.001) (Figure 1, Table 2).

**Figure 2:**
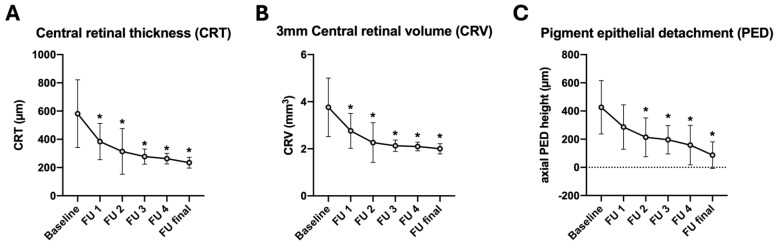
Morphological changes. Significant improvement of the mean central retinal thickness (**A**), 3 mm central retinal volume (**B**) and axial pigment epithelial detachment height (**C**), 4 weeks after triple therapy. Note: FU1: 4 weeks follow-up after intravitreal triple injection; FU2: after first injection series; FU3: 1 year; FU4: 2 years; FU final: Last follow-up examination (after 4.4 ± 1.6 years). * Statistically significant in comparison to baseline measurement; *p* < 0.05.

A notable improvement of all anatomical measures was further observed following triple injection, as summarized in Figure 2. The CRT and CRV thereby both demonstrated immediate and statistically significant improvements, decreasing from 581.1 ± 239.8 µm to 384.1 ± 128.4 µm and from 3.8 ± 1.2 µm^3^ to 2.8 ± 0.7 µm^3^, respectively (*p* < 0.001). In contrast, the axial pigment epithelial detachment (PED) height achieved statistical significance at FU2, following a reduction from 426.3 ± 189.6 µm to 286.0 ± 157.4 µm (*p* < 0.001). The improvements which were observed in all three anatomical measures remained stable until the final follow-up visit (Figure 2, Table 2).

**Figure 3:**
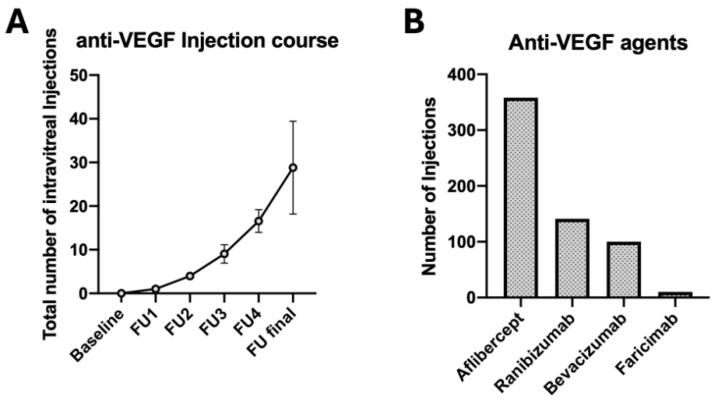
Injection course and distribution of applied anti-VEGF agents. Total number of applied intravitreal injections (**A**). Percentual distribution, Aflibercept: 58.8%, Bevacizumab: 16.4%, Ranibizumab: 23.2%, Faricimab: 1.6% (**B**).

In the first year following triple injection, an average of 9.25 ± 1.9 additional intravitreal anti-VEGF injections were administered due to persistent macular edema. By the end of the second year, the mean total number of injections increased to 16.3 ± 2.6, and at the final follow-up, which was on average 4.4 ± 1.6 years post-triple injection, a cumulative mean number of 29.9 ± 10.9 injections had been administered. The distribution of the applied anti-VEGF agents following triple injection is depicted in Figure 3. The biggest fraction of patients received Aflibercept, followed by Ranibizumab, Bevacizumab, and lastly Faricimab (Figure 3).

In the group comparison of treatment-naïve patients with and without initial SMH, patients with SMH demonstrated a significantly worse mean ETDRS score at baseline, 12 months, and 24 months. Axial PED measurements were also consistently worse in the SMH group across all three time points. In contrast, CRT showed a statistically significant difference only at baseline and after 12 months. Additionally, patients with SMH required a significantly higher number of intravitreal injections during both the first and second year of treatment (Table 3).

The final correlation analysis revealed a positive and statistically significant association between the FU1 VA and the final VA (correlation coefficient: 0.575, *p* = 0.020). On the other hand, no correlation was observed between the final visual acuity and the age of patients, the duration since the onset of symptoms, the size of the subretinal hemorrhage, baseline VA, baseline CRT, or the total number of applied anti-VEGF injections following triple injection (Table 4).

In total, 5 out of 37 eyes (13.5%) experienced a tear of the retinal pigment epithelium following triple injection. The mean IOP was 12.4 ± 4.7 mmHg on the first postoperative day and 13.1 ± 3.5 mmHg on the second postoperative day. Aside from the observed RPE tears, no further adverse events were recorded, such as endophthalmitis, retinal tears, or detachments.

**Figure 4:**
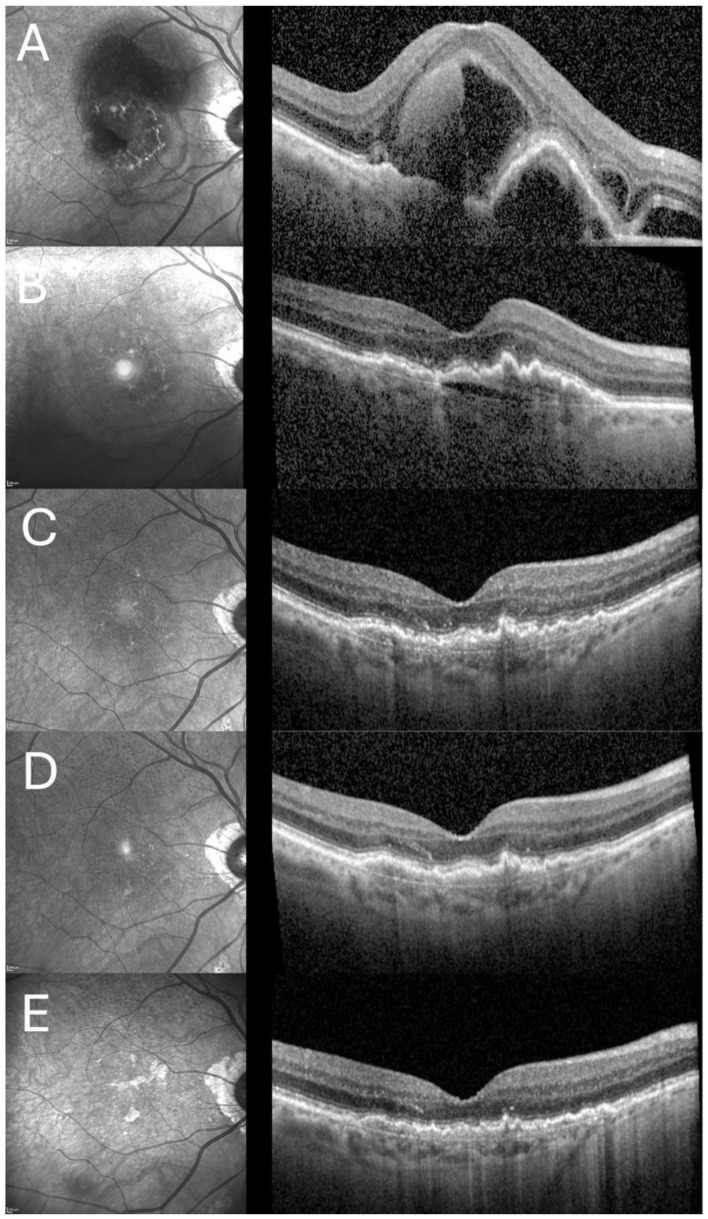
Treatment course of a patient undergoing long-term therapy included in the study: (**A**) Baseline: Presentation 2 days after onset of symptoms, VA: 20 ETDRS letters, CRT: 765 µm; (**B**) Follow-up visit after first anti-VEGF injection series (following 3 anti-VEGF injections), VA: 70 ETDRS letters, CRT: 242 µm; (**C**) Follow-up visit after one year (following 13 anti-VEGF injections), VA: 75 ETDRS letters, CRT: 239 µm; (**D**) Follow-up visit after two years (following 21 anti-VEGF injections), VA: 80 ETDRS letters, CRT: 239 µm; (**E**) Final follow-up visit after 4.8 years (following 43 anti-VEGF injections), VA: 70 ETDRS letters, CRT: 214 µm.

Figure 4 depicts the long-term treatment course of one of the analyzed patients who received triple injection, as well as a subsequent anti-VEGF therapy, due to SMH secondary to age-related macular degeneration.

## 4. Discussion

The mean age of individuals in our study cohort at the time of the hemorrhagic onset was 78 years, which aligns with the average age at which nAMD is typically diagnosed and which is also consistent with prior retrospective studies on SMH secondary to AMD [16,17]. Thereby, most patients (86.5%) in our study cohort were treatment naïve to anti-VEGF therapy prior to the bleeding event, suggesting that hemorrhages during an active anti-VEGF treatment are relatively rare and which is likely attributable to the stabilization of retinal vessels, achieved by intravitreal anti-VEGF therapy. Further, in the first year after triple injection, patients received an average of 9.25 ± 1.9 intravitreal injections due to persistent macular edema, increasing to a total of 16.3 ± 2.6 injections at the end of the second year.

In our comparative analyses of patients treated due to exudative AMD with and without SMH, we observed a notably higher number of anti-VEGF injections among patients with SMH. This increased treatment burden may be attributed to an elevated VEGF release triggered by the bleeding event.

Following the intravitreal injection of a combined triple therapy of rtPA, SF_6_ gas, and Bevacizumab, the further clinical course proved effective in our study. Significant improvements of the ETDRS letter score were observed as early as four weeks after triple injection, with a final gain of +28 letters at the last follow-up visit and in comparison to baseline.

In a retrospective study with a similar treatment approach by de Silva et al., patients affected by SMH secondary to AMD also received a triple injection of 50 μg rtPA, 0.05 mg Ranibizumab, or 1.25 mg Bevacizumab, and 0.3 mL (100%) perfluoropropane (C_3_F_8_) gas [18]. After a mean follow-up period of 7.9 months, patients thereby exhibited notable improvements of + 52 ETDRS letters, from 1.5 ± 61.5 ETDRS letters at baseline to 53.5 ± 68.5 ETDRS letters at the follow-up visit (conversion of LogMAR according to Beck et al. [15]). The baseline VA in the study by de Silva et al. was thereby worse compared to our baseline VA, while the consecutive follow-up VA was comparable to our corresponding FU2 follow-up visit, where patients exhibited a mean ETDRS letter score of 51.5 ± 21.8. Patients in the study cohort of de Silva et al. further exhibited a comparable mean onset of symptom duration of 3.0 ± 1.0 days [18].

Furthermore, in a study by Grohmann et al., patients affected by SMH secondary to AMD were also treated in a similar manner, receiving a triple injection of 20 μg rtPA, 1.25 mg Bevacizumab, and 0.3 mL (16%) hexafluoroethane (C_2_F_6_) gas, followed by a prone positioning protocol, with additional anti-VEGF injections administered in cases of persistent macular edema [19]. Thereby, 6 months after the procedure, patients improved by a mean of +18 ETDRS letters, from 14.5 ± 61 ETDRS letters at baseline to 32.5 ± 59 ETDRS letters during follow-up (conversion of LogMAR according to Beck et al. [15]). In comparison, patients in the study by de Silva et al. and our study showed on average superior improvements after a similar follow-up period. De Silva et al. injected 50 μg rtPA, whereas in our study, 75 μg rtPA was administered intravitreally. In contrast, Grohmann et al. used only 20 μg rtPA, which might be one reason for the difference in the visual outcomes observed. Furthermore, another potential reason could be the longer duration of symptoms in the study by Grohmann et al., with a mean onset of symptoms of 9.1 ± 4.6 days, in comparison to de Silva et al. and our study, as mentioned above. While we did not observe a statistically significant correlation between the symptom duration and the final VA, longer delays in the initiation of treatment could lead to a reduced effect of treatment and worse final VA.

Grohmann et al. also investigated two additional treatment modalities in their study, thereby combining a PPV with an intravitreal injection of 1.25 mg Bevacizumab and 0.3 mL (16%) hexafluoroethane (C_2_F_6_) gas, as well as 20 μg rtPA administered either intravitreally or into the subretinal space. The cohorts that also received a PPV showed no statistical difference in the final visual function compared to the intravitreal triple procedure group without a PPV, and therefore also no superiority compared to our results [19]. Corresponding findings have been described in different meta-analyses, whereby no superiority of a more invasive procedure with a combined PPV and a subretinal rtPA injection was observed in respect to the final visual outcome [10,20].

Regarding morphological changes following triple injection, our study observed a statistically significant improvement in all anatomical measures at six months post treatment. The change of the CRT has also been analyzed in the studies by de Silva et al. and Grohmann et al. and showed comparable improvements [18,19]. Thereby again, regarding the morphological outcomes, and in alignment with the previously reported functional outcomes, Grohmann et al. could not find a distinct difference between the cohorts that also received a PPV or a subretinal rtPA injection [19].

Besides the previously mentioned increased treatment burden, as summarized in Table 3, we further observed that patients with SMH exhibited overall worse functional and morphological clinical measures at baseline, after 12 months, and after 24 months of therapy in comparison to patients without SMH, again highlighting the impact of an initial bleeding event.

Beyond a follow-up duration of 24 months, barely any treatment outcomes of patients affected by SMH have been reported. In one of the few reported studies, Sobolewska et al. treated patients with an intravitreal injection of 50 μg rtPA and 0.5 mL (100%) C_3_F_8_ gas, after a mean onset of symptoms of 8.73 ± 7.4 days [21]. While the baseline mean ETDRS letter score by Sobolewska et al. of 27.5 ETDRS letters was comparable to our baseline VA, the VA reported in the study by Sobolewska et al. improved by 17.5 ETDRS letters to a final score of 45 ETDRS letters after a comparable mean follow-up period of 4.1 years. In direct comparison, in our study, greater improvements were observed, which might again be related to the additional use of an anti-VEGF agent, as well as the shorter symptom duration, as reported above (conversion of LogMAR by Beck et al. [15]). However, in accordance with our results, both studies showed consistent long-term stability following the initial improvements.

No adverse events related to a rise of the IOP have been observed after triple injection, possibly due to the anterior chamber paracentesis that was performed directly after the injection procedure. Still, 13.5% of all treated patients in our study experienced an injection-related complication of a RPE tear. As previously reported in the literature, the single most relevant risk factor associated with tears of the retinal pigment epithelium is the height of the initial RPE detachment [22,23,24,25]. Thereby, as estimated by several studies, detachments of the pigment epithelium exceeding 400–580 microns in height are linked to an increased risk of a RPE tear [22,23]. Further, the overall rate of adverse events following treatment for SMH is difficult to quantify, as it can also partially depend on the operating ophthalmologist, as well as the treatment procedure itself. As estimated by He et al., the rate of adverse events in patients treated for SMH ranges from 2.4% to 20% across intravitreal and subretinal rtPA injection procedures, which aligns in this context with the rate of adverse events observed in our study [9].

The conducted explorative data analysis only revealed a statistically significant association between the 4-week postoperative VA and the final VA. The patient’s age, time since onset of symptoms and size of hemorrhage, baseline VA, baseline CRT, and the number of applied anti-VEGF agents following triple injection showed no association with the final VA. In comparison to the findings by Hattenbach et al. and Sobolewska et al., the duration of an SMH has previously been described as a potential factor influencing final visual acuity. This association is attributed to the damage and degenerative changes in photoreceptor and RPE cells caused by prolonged subretinal hemorrhages [9,21,26]. These studies thereby demonstrated an association in patients treated within the first 14 days after the onset of bleeding. However, since most patients in our study were treated within the first few days after the onset of a SMH with a mean duration of 5.6 ± 5.7 days, it is likely that no statistically significant association was observed. Regarding age and hemorrhage size, no associations have been described in the majority of prior studies [9,26,27], which corresponds with our results. Furthermore, Hattenbach et al. and Sobolewska et al. reported an association between preoperative VA and final VA, which contrasts with our findings [9,21]. Thereby, the lack of a significant correlation in our study may be attributed to the greater improvements observed in our cohort, likely due to the additional use of an anti-VEGF agent.

The biggest strength of our study is the long-term follow-up period, while on the other hand, the small sample size of our cohort as well as the single-center and retrospective design of our investigation constitute limitations. Moreover, visual acuity was initially recorded using the decimal VA, following a conversion to the ETDRS letter score for better comparison, which is inherently less precise.

## 5. Conclusions

In our retrospective study, patients with SMH secondary to AMD who received intravitreal triple therapy with rtPA, SF_6_ gas, and Bevacizumab showed significant and sustained long-term improvements in both visual acuity and morphological outcomes. 13.5% of the patients in our cohort developed a retinal pigment epithelium tear following triple injection, without any other adverse events observed. In comparison to patients treated for nAMD without prior SMH, a higher number of subsequent anti-VEGF injections was applied in the first two years after triple injection, and patients exhibited worse clinical outcomes. The correlation analysis further revealed that postoperative VA 4 weeks after triple injection was predictive for the long-term VA outcomes, while factors such as the preoperative VA, age, hemorrhage size, and the number of applied anti-VEGF injections showed no statistically significant association in our study.

## Figures and Tables

**Table 1:** Baseline characteristics of patient cohort affected by submacular hemorrhage secondary to AMD.

Characteristics	*n* (%)
Number of eyes/Number of patients	37/35
Sex	
male	21 (56.8)
female	16 (43.2)
Age in years (mean ± SD)	78.2 ± 6.8
Laterality	
right	23 (62.1)
left	14 (37.9)
Lens status, phakia/pseudophakia	21/16
Prior treatment history	
Patients having received prior anti-VEGF injections (mean ± SD)	5 (13.5%)
Number of prior anti-VEGF injections (mean ± SD)	21.8 ± 14.3
Days since bleeding event at triple injection (mean ± SD)	5.6 ± 5.7
Size of submacular hemorrhage in disc areas (mean ± SD)	11.4 ± 9.4

Abbreviations: VEGF: Vascular endothelial growth factor.

**Table 2:** Change of visual acuity (VA), central retinal thickness (CRT), axial pigment epithelial detachment height (axial PED), and central retinal volume (CRV).

	Mean ± SD	Difference to Baseline	*p*-Value (Compared to Baseline)
ETDRS baseline	25.6 ± 20.0		
ETDRS FU1	45.4 ± 21.0	+19.8	<0.001 *
ETDRS FU2	51.5 ± 21.8	+25.9	<0.001 *
ETDRS FU3	55.4 ± 19.7	+29.8	<0.001 *
ETDRS FU4	54.2 ± 18.0	+28.6	<0.001 *
ETDRS final	53.8 ± 20.5	+28.2	<0.001 *
CRT (µm) baseline	581.1 ± 239.8		
CRT (µm) FU1	384.1 ± 128.4	−197.0	0.005 *
CRT (µm) FU2	314.0 ± 162.2	−267.1	<0.001 *
CRT (µm) FU3	277.5 ± 53.7	−303.6	<0.001 *
CRT (µm) FU4	263.1 ± 37.6	−318.0	<0.001 *
CRT (µm) final	234.5 ± 38.2	−346.6	<0.001 *
CRV (μm^3^) baseline	3.8 ± 1.2		
CRV (μm^3^) FU1	2.8 ± 0.7	−1.0	0.007 *
CRV (μm^3^) FU2	2.3 ± 0.8	−1.5	<0.001 *
CRV (μm^3^) FU3	2.1 ± 0.2	−1.7	<0.001 *
CRV (μm^3^) FU4	2.1 ± 0.2	−1.7	<0.001 *
CRV (μm^3^) final	2.0 ± 0.2	−1.8	<0.001 *
Axial PED (µm) baseline	426.3 ± 189.6		
Axial PED (µm) FU1	286.0 ± 157.4	−140.3	0.087
Axial PED (µm) FU2	213.4 ± 138.0	−212.9	<0.001 *
Axial PED (µm) FU3	195.9 ± 100.2	−230.4	<0.001 *
Axial PED (µm) FU4	158.2 ± 87.0	−268.1	<0.001 *
Axial PED (µm) final	140.4 ± 94.4	−285.9	<0.001 *

Abbreviations: CRT: central retinal thickness; CRV: central retinal volume; PED: pigment epithelial detachment. Note: * Statistically significant in comparison to baseline measurement; *p* < 0.05.

**Table 3:** Univariate analysis of treatment outcomes in treatment-naïve eAMD patients with and without acute submacular hemorrhage.

	eAMD Without SMH (*n* = 32)	eAMD with SMH (*n* = 32)	*p*
Age (years) at first treatment (mean ± SD)	75.8 ± 6.8	77.9 ± 7.0	0.09
Baseline ETDRS score (mean ± SD)	65.5 ± 11.9	27.8 ± 19.9	<0.001 *
12. months ETDRS score (mean ± SD)	69.8 ± 12.1	46.4 ± 20.0	<0.001 *
24 months ETDRS score (mean ± SD)	69.1 ± 9.5	53.2 ± 20.9	<0.001 *
Baseline CRT (mean ± SD)	342.0 ± 69.7	571.3 ± 236.0	<0.001 *
12 months CRT (mean ± SD)	286 ± 57.2	388.1 ± 137.9	0.015 *
24 months CRT (mean ± SD)	268.9 ± 55.6	309.7 ± 164.1	0.11
Baseline axial PED (mean ± SD)	147.0 ± 119.4	448.2 ± 178.3	<0.001 *
12 months axial PED (mean ± SD)	117.7 ± 78.6	320.9 ± 153.1	0.002 *
24 months axial PED (mean ± SD)	101.6 ± 63.2	217.9 ± 139.3	0.001 *
Nr. of IVI after 12 months (mean ± SD)	7.6 ± 1.6	9.4 ± 1.8	<0.001 *
Nr. of IVI after 24 months (mean ± SD)	14.4 ± 2.9	16.7 ± 2.1	0.019 *

Note: Baseline = before treatment; FU1 = 12-month exam; FU2 = 24-month exam; * = *p* < 0.05. Abbreviations: CRT: central retinal thickness; PED: pigment epithelial detachment; IVI: intravitreal injection.

**Table 4:** Explorative data analysis on final visual function.

	Correlation Coefficient	*p*-Value
Age	−0.322	0.224
Days since hemorrhage	−0.048	0.876
Size of subretinal hemorrhage (disc areas)	−0.365	0.269
Visual acuity baseline (ETDRS)	0.320	0.227
Visual acuity 4 weeks after triple injection (ETDRS)	0.575	0.020 *
Central retinal thickness baseline (µm)	0.190	0.554
Number of anti-VEGF injections after event	−0.141	0.603

Note: Pearson correlation, * *p* < 0.05.

## Data Availability

The data underlying this manuscript are not publicly accessible due to privacy constraints, with the exception of data already officially published.
